# Analysis of Serum Vitamin D Level and Related Factors in Patients With Restless Legs Syndrome

**DOI:** 10.3389/fneur.2021.782565

**Published:** 2021-12-09

**Authors:** Hui Miao Liu, Miao Chu, Chen Fei Liu, Ting Zhang, Ping Gu

**Affiliations:** The First Hospital of Hebei Medical University, Shijiazhuang, China

**Keywords:** restless legs syndrome, vitamin D, severity of symptoms, sleep quality, depression

## Abstract

**Objective:** This study aimed to detect serum vitamin D (VitD) levels in patients with primary restless legs syndrome (RLS). The further objective was to analyze the relationship of VitD levels with the severity of RLS symptoms, sleep, anxiety, and depression.

**Methods:** The serum 25-hydroxyvitamin D [25(OH)D] levels of 57 patients with primary RLS and the healthy physical examinees in our hospital during the same period were detected. The International Restless Legs Syndrome Study Group (IRLSSG) rating scale for measuring RLS severity and Pittsburgh Sleep Quality Index (PSQI) Scale, 24-item Hamilton Depression Rating Scale (HAMD_24_), and 14-item Hamilton Anxiety Scale (HAMA_14_) were used to assess the severity of symptoms, sleep, and emotional state of patients with RLS. Based on VitD level and IRLSSG score, they were grouped for analysis.

**Results:** The serum 25(OH)D level was significantly lower in patients with RLS than in healthy controls, and the incidence of insufficient serum VitD levels was significantly higher in patients with RLS than in healthy people (both *P* < 0.05). The serum VitD level was significantly lower in (extremely) severe patients with RLS than in mild to moderate patients with RLS (*P* < 0.05). The IRLSSG scale score and HAMD_24_ score were significantly higher in patients with RLS with insufficient serum VitD levels than those with normal serum VitD levels (both *P* < 0.05). Correlation analysis of IRLSSG scale score with serum VitD level and each scale score in patients with RLS showed that IRLSSG scale score was negatively correlated with VitD level, but positively correlated with PSQI, HAMA_14_, and HAMD_24_ scores. The results of correlation analysis between serum VitD levels and each scale score in patients with RLS indicated that serum VitD levels were negatively correlated with IRLSSG scale scores, PSQI scores, and HAMD_24_ scores.

**Conclusion:** The serum VitD level is generally lower in patients with RLS than in healthy people, and lower serum VitD level is associated with more severe symptoms of RLS, worse quality of sleep, and worse depression.

## Introduction

Restless legs syndrome (RLS) is one of the most common sleep-related sensorimotor disorders of the central nervous system, which is mainly manifested as indescribable limb discomfort, such as “prickly or stinging sensation,” “creeping sensation,” and “burning sensation” in the resting state, especially before going to bed or during quiet rest. It can occur on one side or both sides of the limbs, specifically in the lower limbs. The above limb discomfort symptoms are usually unbearable, forcing patients to move or massage their limbs to relieve the discomfort symptoms (1). The prevalence of RLS is approximately 0.1–15% (2–4). Secondary RLS can be secondary to chronic kidney disease, iron deficiency anemia, diabetes, pregnancy, Parkinson's disease, and multiple sclerosis, etc.

At present, relevant studies mainly focus on brain iron deficiency, neurotransmitter regulation disorders including dopaminergic system, and genetic factors. Brain iron deficiency is considered to be the key pathogenesis, which is jointly affected by environmental factors and genetic inheritance (5). Vitamin D (VitD) is involved in regulating the activity of the amygdala to improve patients' depression and anxiety.

Recent studies have reported (6–8) the presence of generally insufficient serum VitD levels in patients with primary RLS. This clinical retrospective study aimed to explore the correlation of the serum VitD level with the severity of RLS symptoms, sleep quality, anxiety, and depression in patients with primary RLS, so as to provide a basis for the prevention and treatment of RLS.

## Materials and Methods

### Research Subjects

A total of 57 primary patients with RLS that were treated in the Department of Neurology, the First Hospital of Hebei Medical University from October 2019 to October 2020 were enrolled in this study. Another 57 age- and gender-matched healthy people who underwent physical examinations during the same period were selected as the control group.

#### Inclusion Criteria

Patients that conformed to the diagnostic criteria of RLS. The consensus by the International Restless Legs Syndrome Study Group (IRLSSG) in 2014 proposed new diagnostic criteria of RLS/Willis-Ekbom disease (WES) (9).

#### Exclusion Criteria

(1) RLS secondary to chronic kidney disease, renal failure, iron deficiency anemia, pregnancy, peripheral neuropathy, Parkinson's disease, and Parkinson's syndrome, drug-induced factors, including taking anti-depressants or sedatives and sleeping pills, familial and genetic factors; (2) other sleep disorders such as rapid eye movement sleep behavior disorder, sleep apnea syndrome, etc.; (3) metabolic syndrome, osteoporosis, cerebrovascular disease, respiratory diseases, malignant tumors, thyroid diseases, various mental diseases, liver and kidney diseases, and other diseases that affected serum VitD levels; (4) patients taking calcium, calcidiol capsules, and other drugs that affected serum VitD levels or drugs that alleviated RLS; (5) pregnant and lactating women. This study was approved by the Ethics Committee of First Hospital of Hebei Medical University, and written informed consent was obtained from the enrolled patients.

### Research Methods

#### Collecting General Information

The information such as the name, gender, age, chief complaint, present medical history, and previous underlying diseases of the patients and healthy controls was collected.

#### Evaluation of Symptoms, Sleep Quality, and Mood of the Patients

Professional physicians inquired about RLS symptoms, severity, sleep quality, anxiety, and depression of patients with RLS, and conducted corresponding scale assessments, including the IRLSSG rating scale for measuring RLS severity, Pittsburgh Sleep Quality Index (PSQI) scale, 24-item Hamilton Depression Rating Scale (HAMD_24_), and 14-item Hamilton Anxiety Scale (HAMA_14_).

#### Detection of Serum VitD Level

Early morning fasting venous blood was collected from patients with RLS and healthy controls, and magnetic particle chemiluminescence immunoassay was used to detect serum 25-hydroxyvitamin D [25(OH)D] levels.

#### Grouping

According to the serum 25(OH)D level of patients with primary RLS, they were divided into normal VitD level [25(OH)D ≥ 20 ng/ml] group and insufficient VitD level [25(OH)D <20 ng/ml] group. According to the severity of limb discomfort (10, 11), patients with RLS were divided into mild to moderate RLS group (IRLSSG: 1–20 points) and (extremely) severe RLS group (IRLSSG: 21–40 points).

### Statistical Analysis

The statistical software SPSS 25 (IBM, Chicago) was used for statistical analysis. The measurement data conformed to normal distribution were expressed as the mean ± *SD*, and the independent sample *t*-test was used for the comparison between groups. The measurement data of non-normal distribution were expressed as the median and interquartile range, and the Mann-Whitney U-test was used for comparison between groups. The measurement data were expressed by the number of cases (percentage) method, and the comparison between groups was performed by χ^2^-tests. Pearson correlation analysis was used for analysis between serum VitD level and IRLSSG scale score. *P* < 0.05 indicated that the difference was statistically significant.

## Results

### Baseline Information

A total of 57 patients with RLS were enrolled in this study, including 19 men (33.33%) and 38 women (66.67%). The ratio of men to women patients with RLS was 1:2. The age ranged from 15 to 78 years, with a mean age of (57.56 ± 13) years. A total of 57 cases were enrolled in the healthy control group, including 17 men (29.82%) and 40 women (70.18%), aged 18–79 years, with a mean age of (57.68 ± 12.9) years. No significant difference was found in age and gender between the two groups (*P* > 0.05). The serum VitD level was lower in patients with RLS than in the healthy controls, and the incidence of insufficient serum VitD level was significantly higher in primary patients with RLS than in healthy people (both *P* < 0.05) (see Table 1).

**Table 1:** Comparison of gender, age, and VitD level between RLS group and healthy control group.

	**Primary RLS group (*n* = 57)**	**Healthy control group (*n* = 57)**	***F*/χ^2^**	***P***
Gender (female/male)	38/19	40/17	0.162	0.687
Age (*$\bar{\chi }$* ±*S*, years)	57.56 ± 13.00	57.68 ± 12.90	0.049	0.960
VitD level (*$\bar{\chi }$* ±*S*, ng/ml)	16.07 ± 5.43	27.00 ± 5.00	0.224	0.000^*^
Incidence of insufficient VitD level (*n*, %)	46 (80.70%)	1 (1.57%)	73.309	0.000^*^

* *P <0.05*.

### Comparison of Clinical Data of Patients With Mild to Moderate RLS and Those With (Extremely) Severe RLS

Among 57 patients with RLS, 36 patients (63.16%) had mild to moderate RLS, including 12 men and 24 women aged 31–74 years, with a mean age of (56.81 ± 12.33) years. There were 21 (36.84%) (extremely) severe patients with RLS, including 7 men and 14 women aged 15–78 years, with a mean age of (58.86 ± 14.28) years. No significant difference was found in gender and age between the two groups (*P* > 0.05). The serum VitD level of (extremely) was significantly lower in severe patients with RLS than in mild to moderate patients with RLS (*P* < 0.05) (see Table 2).

**Table 2:** Comparison of clinical data between mild to moderate RLS patient group and (extremely) severe RLS patient group.

	**Mild to moderate RLS group**	**(Extremely) severe RLS group**	***F***	***P***
No. of cases (*n*, %)	36 (63.16%)	21 (36.84%)		
VitD level (*$\bar{\chi }$* ±*S*, ng/ml)	17.26 ± 5.44	14.04 ± 4.88	0.121	0.029^*^
PSQI (*$\bar{\chi }$* ±*S*, 分)	15.92 ± 3.73	16.19 ± 2.07	6.404	0.758
HAMA_14_ (*$\bar{\chi }$* ±*S*, 分)	15.19 ± 5.12	16.71 ± 5.08	0.744	0.283
HAMD_24_ (*$\bar{\chi }$* ±*S*, 分)	16.22 ± 6.14	17.71 ± 6.91	0.075	0.402

* *P <0.05*.

### Comparison of RLS Severity, Sleep Quality, and Anxiety and Depression in Normal Serum VitD Level Group and Insufficient VitD Level Group

Among 57 patients with RLS, 11 patients (19.3%) were in the normal VitD level group, including 5 men and 6 women aged 38–69 years, with a mean age of (59.36 ± 9.1) years; 46 patients (80.7%) were in the VitD level group, including 14 men and 32 women aged 15–78 years, with a mean age of (57.13 ± 13.81) years. There was no statistical difference in gender and age between the two groups (*P* > 0.05). The IRLSSG scale score and HAMD_24_ score of patients with RLS were significantly higher in the insufficient serum VitD level group than in the normal serum VitD level group (both *P* < 0.05) (see Table 3).

**Table 3:** Comparison of clinical data between the normal VitD level group and the insufficient VitD level group.

	**Normal VitD level group**	**Insufficient VitD level group**	***Z/F***	***P***
No. of cases (*n*, %)	11 (19.3%)	46 (80.7%)		
IRLSSG [*M*(*P*_25_, *P*_75_), points]	14 (11, 18)	19 (15.75, 23)	−2.147	0.032^*^
PSQI (*$\bar{\chi }$* ±*S*, points)	14.91 ± 4.46	16.28 ± 2.82	3.352	0.204
HAMA_14_ (*$\bar{\chi }$* ±*S*, points)	15.09 ± 6.36	15.91 ± 4.83	2.619	0.636
HAMD_24_ (*$\bar{\chi }$* ±*S*, points)	12.82 ± 5.47	17.72 ± 6.31	0.240	0.021^*^

* *P <0.05*.

### Correlation Analysis of IRLSSG Score With Serum VitD Level and Various Scale Scores in Patients With RLS

Pearson correlation analysis showed that IRLSSG score was negatively correlated with serum VitD level (*r* = −0.395, *P* = 0.002), but positively correlated with PSQI score, HAMA_14_ score, and HAMD_24_ score (*r* = 0.374, *P* = 0.004, *r* = 0.348, *P* = 0.008, *r* = 0.347, *P*= 0.008) (see Table 4).

**Table 4:** Pearson correlation analysis of IRLSSG score with serum VitD level and various scale scores.

	***r***	***P***
VitD level	−0.395	0.002^*^
PSQI	0.374	0.004^*^
HAMA_14_	0.348	0.008^*^
HAMD_24_	0.347	0.008^*^

* *P <0.05*.

### Correlation Analysis Between Serum VitD Level and Various Scale Scores in Patients With RLS

Pearson correlation analysis indicated that serum VitD level was negatively correlated with IRLSSG score, PSQI score, and HAMD_24_ score (*r* = −0.395, *P* = 0.002, *r* = −0.304, *P* = 0.022, *r* = −0.295, *P* = 0.027) (see Table 5).

**Table 5:** Pearson correlation analysis between the VitD level of patients with RLS and various scale scores.

	***r***	***P***
IRLSSG	−0.395	0.002^*^
PSQI	−0.304	0.022^*^
HAMD_24_	−0.294	0.027^*^

* *P <0.05*.

## Discussion

Vitamin D participates in the synthesis and release of neurotransmitters, and plays an anti-inflammatory, anti-oxidant, and neuroprotective role, exerting an important effect on regulating the occurrence and development of sleep, anxiety, and depression (12, 13). Vitamin D deficiency can lead to reduced levels of dopamine and its metabolites in the brain, and the decreased release of dopamine in the substantia nigra of the midbrain and dopaminergic transmission disorders are common pathogenesis of RLS. Multiple studies have found that VitD deficiency is common in patients with RLS. A cross-sectional study of 102 patients with RLS by Cakir et al. (5) found that the incidence of VitD deficiency was higher in patients with RLS (52.63 vs. 37.78%), and the PSQI scores of patients with VitD deficiency were higher (*P* < 0.05); patients with RLS with VitD deficiency had more frequent symptoms of limb discomfort. Almeneessier et al. (14) studied 1,136 non-pregnant women with RLS in Saudi Arabia and found that VitD deficiency was an independent risk factor for RLS [odds ratio (OR): 2.147 (1.612–2.86), *P* < 0.001]. A meta-analysis of 9,590 patients with RLS showed (15) that there was a significant correlation between serum VitD levels and RLS (95% Cl: −5.96 to −0.81, *P* = 0.01, *I*^2^ = 86.2%). This study also found that patients with primary RLS had low serum VitD levels, and the incidence was higher in patients with insufficient VitD levels than in healthy people.

Restless leg syndrome occurs at any age, but the prevalence of RLS is higher in women than in men; and the incidence of RLS in women is approximately twice that of the general population (16, 17), which may be related to low serum iron and ferritin levels during pregnancy, high estrogen levels, VitD deficiency and calcium metabolism disorders (18, 19). A prospective case-control study by Balaban et al. (20) showed that the average serum VitD level was lower in female patients with RLS than in patients who are men and that there was a significant negative correlation between female VitD levels and the severity of RLS disease (*r* = −0.47, *P* = 0.01). In this study, the ratio of men to women patients with RLS was 1:2. However, there was no difference in VitD levels between male and female patients, which might be related to the small sample size in this study.

Serum VitD levels were lower in patients with (extremely) severe RLS than in those with mild to moderate RLS, which were negatively correlated with IRLSSG scores. The results of this study suggested that more severe symptoms of limb discomfort in patients with RLS were associated with lower serum VitD levels. Therefore, it is controversial whether correcting insufficient serum VitD levels can improve the symptoms of limb discomfort in patients with RLS. Wali et al. (21) recommended VitD supplement treatment to patients with primary RLS and insufficient VitD levels. When the VitD level was adjusted to >50 nmol/L, the severity of RLS was evaluated and compared with the score before taking the supplement. It was found that the IRLSSG score was significantly decreased (*P* = 0.002). This preliminary study revealed that drug supplementation with VitD could ameliorate the severity of RLS symptoms. Tutuncu et al. (7) conducted a prospective self-controlled case study to treat 21 patients with RLS with insufficient VitD levels by taking 50,000 units of VitD supplements per week for 2 months. After 2 months, the patients had normal VitD levels, and some of the IRLSSG scores were also improved, including the severity of symptoms (*P* < 0.001), the impact on sleep (*P* < 0.001), the assessment of symptoms (*P* = 0.002), and the assessment of the impact of disease on life (*P* < 0.001). There was a trend of improvement in the two sub-items of seizure frequency (*P* = 0.11) and mood (*P* = 0.051). Nonetheless, several opposite conclusions were also drawn from this study. Wali et al. (22) conducted a 12-week randomized placebo-controlled trial and found that compared with the placebo group, 35 subjects who received oral 50,000 IU VitD capsules experienced no significant changes in the severity of RLS symptoms. The results showed that VitD supplementation failed to alleviate the symptoms of RLS. It is inconclusive whether drugs supplemented with VitD are effective in the treatment of RLS. Long-term, large-sample prospective cohort studies, and clinical trials are still needed to support this relationship and evaluate the efficacy of VitD for the treatment of RLS. However, for patients with RLS with insufficient VitD levels, regular exposure to sunlight, outdoor exercise, and dietary supplementation with VitD are all recommended.

Patients with RLS often experience various discomforts such as limb pain, swelling, burning, creeping sensation, which can lead to difficulty falling asleep and sleep interruption. Patients with RLS may suffer daytime sleepiness, fatigue, inattention, and decreased interest, depression, irritability, and other anxiety and depression emotions, affecting the quality of sleep and quality of life of patients (2, 23–25). Vitamin D regulates the production of melatonin in the body and plays an important role in regulating the circadian rhythm of sleep. In addition, VitD is associated with increased levels of γ-aminobutyric acid, glutamine, and glutamate in the hippocampus, prefrontal cortex, and anterior cingulate gyrus, which may help regulate depressive mood and anti-anxiety effects (26, 27). A retrospective study by Atalar et al. (28) showed that compared with patients with RLS with normal VitD levels, patients with RLS with VitD deficiency had worse sleep quality (*P* < 0.05). Turk et al. (29) reported that 91.9% of patients with RLS had prolonged sleep latency, poor sleep quality, short sleep duration, and daytime dysfunction. Patients with moderate to severe RLS had a higher incidence of night sleep disturbance, daytime fatigue, and depression (16). Yilmaz et al. (30) found that the RLS severity score was positively correlated with the anxiety and depression scale scores (*P* < 0.05). The probability of depression in patients with RLS was 2.5–5 times higher than that in patients without RLS, and the probability of depression and the severity of insomnia both increased with the worsened severity of RLS symptoms (31). This study found that the worse symptoms of patients with RLS led to the worse sleep quality, and the more severe anxiety and depression accompanied. In addition, the lower serum VitD level of patients with RLS was associated with the more severe RLS symptoms, the worse quality of sleep, and the worse depression.

In conclusion, VitD deficiency is more common in patients with RLS. The lower the serum VitD level in patients with RLS, the more severe the symptoms of limb discomfort in patients with RLS, the worse the quality of sleep, and the worse the depression. Clinicians should pay attention to the detection of serum VitD levels and the evaluation of anxiety and depression during the diagnosis and treatment of RLS. However, whether VitD deficiency will cause the occurrence of RLS and the aggravation of symptoms as well as their causal relationship remains to be further studied. Conventional drug supplemented with VitD as an intervention for RLS is still controversial, and a large number of long-term prospective cohort studies and clinical trials are needed to confirm the findings. However, dietary supplementation with VitD, regular exposure to sunlight, and outdoor exercise is recommended for patients with RLS. Given that this is a clinical retrospective study with a small sample size, multi-center, large-sample clinical studies are needed in the future to further improve placebo-controlled study and explore the changes in RLS symptoms, mood, and sleep quality before and after supplementation with VitD. In our next study, we will aim to analyze the correlation between vitD level and RLS symptoms by age.

## Data Availability Statement

The original contributions presented in the study are included in the article/supplementary material, further inquiries can be directed to the corresponding author/s.

## Ethics Statement

The studies involving human participants were reviewed and approved by the First Hospital of Hebei Medical University. The patients/participants provided their written informed consent to participate in this study.

## Author Contributions

All authors listed have made a substantial, direct, and intellectual contribution to the work and approved it for publication.

## Conflict of Interest

The authors declare that the research was conducted in the absence of any commercial or financial relationships that could be construed as a potential conflict of interest.

## Publisher's Note

All claims expressed in this article are solely those of the authors and do not necessarily represent those of their affiliated organizations, or those of the publisher, the editors and the reviewers. Any product that may be evaluated in this article, or claim that may be made by its manufacturer, is not guaranteed or endorsed by the publisher.
